# Are children under five with disabilities' educational rights acknowledged and supported in Chile?

**DOI:** 10.3389/fpubh.2024.1304152

**Published:** 2024-03-15

**Authors:** Mauricio López, Andrea Mira, Daniela Mauricia, Paulina Acevedo, Ruth Lopez, Pamela Molina, Lina Gutiérrez, Eloisa de Billerbeck, Cecilia Breinbauer

**Affiliations:** ^1^Millennium Nucleus Studies on Disability and Citizenship (DISCA), Santiago, Chile; ^2^Exercise and Rehabilitation Sciences Institute, School of Occupational Therapy, Faculty of Rehabilitation Sciences, Universidad Andres Bello, Santiago, Chile; ^3^Department of Child and Adolescent Development, Center for Healthy Development, Round Hill, VA, United States; ^4^World Federation of the Deaf, Helsinki, Finland; ^5^Arica Autism Association, Arica, Chile

**Keywords:** children with disabilities, developmental disabilities, early childhood development, early intervention, inclusive education

## Introduction

In Chile last May 2023, the Ministry of Education in the webinar “Role of the Autism Spectrum Disorder (ASD) Law^1^ in the current regulatory framework” released revealing data indicating that in 2015, 3,731 autistic students were registered in the School Integration Program (Programa de Integración Escolar—PIE), a number that in 2023 reached 43,428 autistic students, an increase of more than 1,000% in 8 years (1). PIE is an inclusive strategy of the educational system, which has the purpose of contributing to the continuous improvement of the quality of education, favoring learning in the classroom and the participation of each and every one of the students, especially those with Special Educational Needs (SEN) (2). But not all schools have PIE. The coordinator for attention to the diversity of the Ministry of Education, in this same webinar, said that there is no data on the participation of autistic students in educational establishments that do not have PIE or in nursery education, so these statistics may even be more substantial and do not consider under-five children with disabilities.

It is crucial to optimize school readiness for inclusive and equitable quality education for the most vulnerable children (3). Children with disabilities usually experience social and educational exclusion with an essential impact on their mental health and wellbeing (3). For this reason, it is relevant to have efficient early development screening and follow-up systems, adequate records, and the design of support systems that respond to children's needs and their families. In this way, the initiatives for enhancing early child development should prioritize children with developmental disabilities, nevertheless, this requires a multi-sectoral coordination that favors adequate indicators, monitoring, and evaluation of policies and services delivered.

States around the world generate a large amount of data on the management and governance of the country. Historically, much of this data in Chile was only accessible through statistical reports that were not easily accessible to the public. In 2009, Chile enacted a law related to facilitating access to public information (4), and created an autonomous institution and guarantor of this regulation, the Council for Transparency (CPLT—Consejo para la Transparencia). Since then, any person in Chile has the right to request information from state administration departments, who, in turn, have the duty to respond to this requirement (5). The information that can be requested is the one related to the acts and resolutions of the State administration bodies, their foundations, the documents that serve as support, and the procedures used for their issuance. All information that is prepared with a public budget, whatever the format or medium in which it is contained, except for the exceptions contemplated in the Transparency Law. However, the technical feasibility and interconnection of the different institutions in charge of delivering the information are still lacking. Most of the information exists, but it is up to individuals and institutions how and what information they share with the general public.

## Chilean national disability registration: available information regarding young children?

On August 25, 2008, Chile promulgated the decree 201 (6) ratifying the Convention on the Rights of Persons with Disabilities (CRPD) of the United Nations (UN). On February 10, 2010, Law No. 20,422 came into effect, establishing rules on equal opportunities and social inclusion of people with disabilities (7). Article 31 of the CRPD establishes that States parties must collect adequate information that allows them to formulate and implement public policies that ensure compliance with the Convention. This, in conjunction with Chilean Law No. 20.422 (7), establishes that the only way to accredit the disability and access its social benefits is through registration in the National Registry of Disability (RND—Registro Nacional de Discapacidad) (7). This requires completing the Disability Qualification and Certification process, which conforms to the guidelines and standards of the International Classification of Functioning (ICF) of the World Health Organization (WHO).

Although registration in the RND is voluntary in Chile, People with Disabilities (PWD) are encouraged to join. Having the information of all -or most- PWDs would be an important source of information for formulating public policies according to their needs. Having a reliable source of statistical information would be a major success for the country because the statistics now are simply referential. Unfortunately, there is currently no public information on how many families with children under five with disabilities have completed the process of obtaining the disability credential, a physical document that is used daily to access benefits for the person who is enrolled in the RND. The only sources of public information are the National Survey of Health and Disability (Encuesta Nacional de la Discapacidad, ENDISC) (8) and the National Disability and Dependency Survey (Encuesta Nacional de la Discapacidad y Dependencia, ENDIDE) (9).

As stated in ENDISC and ENDIDE, 17.6% of the Chilean population over 18 has a disability, while 14.7% are children between 2 and 17 years old. According to our estimates, using the information from this survey and the information provided by SENADIS, about 12.7% of them would be children between 2 and 5 years old. Among 5.526 children between 2 and 17 years old, only 94 (1.7%) are enrolled with the RND. In Colombia, for example, the Registry for the Location and Characterization of People with Disabilities (RLCPD) registered 981,181 PWD as of May 2013, a number that, to date, corresponds to 37.4% of PWD identified in the Census (10).

To have a better understanding of the information that different sectors have regarding under-five children with disabilities in Chile, we requested, through the CLPT, additional reports to six Chilean governmental institutions that provide services to young children:

1. National Disability Service (SENADIS).
2. Civil Registry and Identification Service (SRCEI).
3. Sub-Secretariat of Nursery Education.
4. Integra Foundation.
5. National Board of Kindergartens (JUNJI).
6. Sub-Secretariat of Health.

The institutions that were contacted answered the request for information within the time indicated by the law. However, we received only partial, fragmented information, not disaggregated by age. We did not receive annual reports of students enrolled within the school system who are also enrolled in the RND from any of the institutions we requested information from.

## Data sources on institutional intersectionality

With < 2% of the children between 2 and 17 years old registered on the RND, Chile has a massive debt with under-registration (9). Furthermore, no Chilean governmental institution seems to publish annual reports tracking both school enrollment and disability. Specifically, we requested the last five national annual reports of students enrolled within the school system who are also enrolled in the RND, disaggregated by age (0–5/6–12/13–18 years), region, and type of disability. Law No. 20.422, in article 55 (7), says that the RND is an administrative registry dependent on the *SRCEI*. So, we decided to ask first to the *SRCEI*, but they said that the institution in charge of that information is *SENADIS*. In parallel, we asked for data from the *Sub-Secretariat of Nursery Education* and the *Sub-Secretariat of Health*, the main two ministers that could be involved.

**Figure 1:**
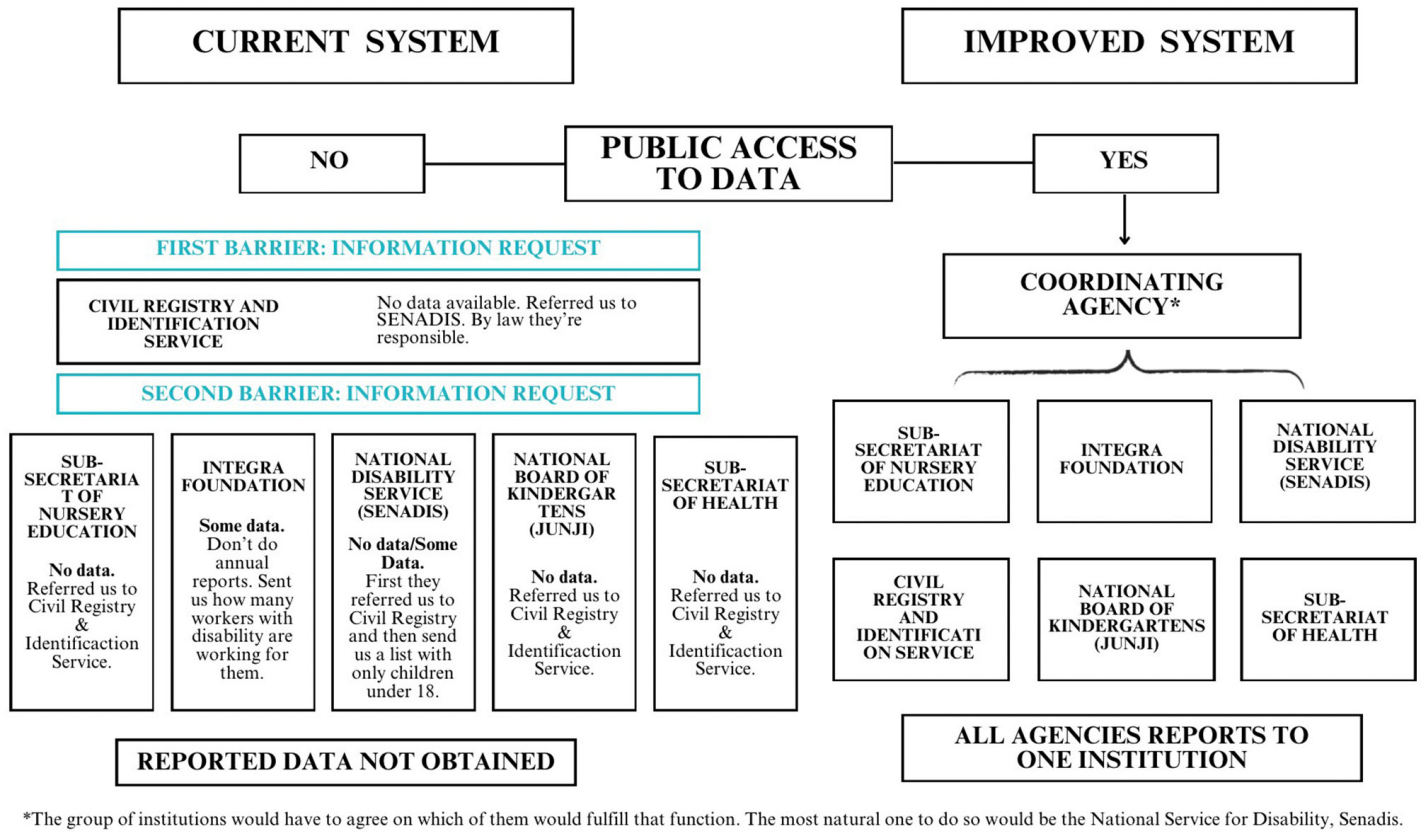
This figure shows a simplified representation of how the system currently works regarding access to public information. The right side of the figure shows a proposal for how the system could be organized to promote better access to information. ^*^The group of institutions would have to agree on which of them would fulfill that function. The most natural one to do so would be the National Service for Disability, Senadis.

The first one was categorical to answer that they don't have that type of information and deriving us fully to *SENADIS*, again, and the second also categorically answer that they don't have that type of information, but derive us fully to the *SRCEI*. This was the first contradiction that the different agencies had about existing information. We also try to get data from *JUNJI* and *INTEGRA*, both governmental institutions that provide public nursery education. *JUNJI* once again referred us to the *SRCEI* and *INTEGRA* answered that they don't do annual reports, and sent us an archive of the workers with disability working for *INTEGRA*. *SENADIS* did reply with the information we solicited, but they didn't specify children under-five with disabilities, only children under 18. We can see here that there is no interconnectivity between government agencies, and there is no clear view of which institution is responsible for it. The public information available seems to indicate that the RND does not support the educational rights of children under five in Chile (See Figure 1).

## Recommendations

During this investigation, we could not identify nor get information from official sources that can currently be used to determine how children under five with disabilities are being supported in their future endeavors to get into the regular educational system. As mental health and disability researchers, almost half of us being People with Disabilities and an autistic researcher, we are concerned about the absence of information available and the interconnectivity between governmental agencies to this issue. Based on the results of this investigation, our recommendations are as follows (Figure 1):

- Although the RND is an administrative registry, supposedly dependent on the *SRCEI*, we couldn't get any information from this agency. They even argue that the institution in charge of the registry was *SENADIS*. The lack of interconnectivity between governmental agencies and the absence of acknowledgment of the administration of the registry makes it very difficult for the general public and congressmen and women to have serious and ready-to-access statistical data to make conscious legislation about this topic. Having only one institution that manages the registry may ensure reliable information that could lead to relevant and suitable legislation for PWD and, especially, educational laws that optimize school readiness for inclusive and equitable quality education for children with disabilities.
- While the RND is voluntary, it does offer a series of benefits to families with young children who have a disability, such as preferential access to health services, access to specialized equipment and other technical support, open choice therapy, and others. The under-registration in the RND must be addressed with new strategies that warrant anonymity, scholar and work-related inclusion, and the benefits of being part of it. People must feel drawn to be part of the registry.
- It is worrying that there are no apparent records of the number of children under 2 years of age with disabilities, so it is challenging to design appropriate policies and interventions if there is no precise data about this group. This is critical, especially when considering preterm birth and the risk these children have to present significant neurodevelopmental disabilities, like cerebral palsy, visual impairment, intellectual disabilities, and hearing loss (11). Moreover, they can also present other neurodevelopmental difficulties with executive functions, academic underachievement, and problems with motor coordination, among others (12). Not having these statistical data might harm the distribution of the annual governmental budget and the consideration of the PWD community. Having a more age-inclusive policy for the registry may help ensure a scholar budget and have a more robust inclusion team in PIEs along the country, acknowledging every region's challenges.
- Data on the prevalence of congenital hearing loss in Chile have not been established. Figures published by the National Commission for Monitoring of Premature Births show a prevalence of Hearing loss in children under 1,500 g of 3.4%, 60–80 cases per year. Even though the government must have data on the children born with a hearing disability because of the Hearing Treatment policy for Moderate, Severe, and Deep Hearing Loss of children under 4 years by the Ministry of Health, there is no official record of them. Knowing about the children with hearing loss could help policies that guarantee Chilean Sign Language as a main priority in the scholar curriculum.

## Conclusion

The process of inclusion in Chile starts with Decree 83 (13), a diversification of teaching from 2015 that approves criteria and guidelines for curricular adequacy for students with SEN in preschool and elementary education. The same year, legislators ratified Law No. 20.845 (14) of school inclusion, which regulates the admission of students and, eliminates shared financing, and prohibits profit in educational establishments that receive the State's financial support. Law No. 21.545 (15), which establishes the promotion of inclusion, comprehensive care, and the protection of the rights of autistic people in the social, health, and educational fields, reinforces the Chilean government's commitment to inclusion. Unfortunately, we faced difficulties accessing information that can help us understand how Chilean government institutions detect and register data related to children with disabilities. We can acknowledge legislation, but it does not relate to the registry information or agencies' statistics.

This difficulty in accessing accurate information does not mean there are no policies or early services delivered by the health, education, or social protection sectors. However, it is necessary to have more accurate information about children's needs and the support that the government displays. Access to public information promotes an enrichment of democracy by strengthening the supervision of government entities. In addition, this is expected to raise the quality of citizen engagement, modernize the state administration, and constitute a human right for informed decision-making for life and correct citizen participation (16). Our brief review of the matter for this article highlights how young children with disabilities (0–5 years old) are still not given priority to support their educational needs. Children must be a priority, and this should reflect a change in political priorities and mobilize resources, ensuring the effective delivery and monitoring of services. To accelerate progress toward the achievement of an inclusive, equitable, and quality education and promote learning opportunities during all life for all people, children under 5 years old with developmental disabilities should also be the focus of public policies from birth because of the importance of early detection and interventions. Governments and the community can work together so no child is left behind.

## Author contributions

ML: Conceptualization, Investigation, Writing—original draft, Writing—review & editing. AM: Conceptualization, Investigation, Methodology, Supervision, Writing—original draft, Writing—review & editing. DM: Writing—review & editing. PA: Writing—review & editing. RL: Writing—review & editing. PM: Writing—review & editing. LG: Writing—review & editing. EB: Writing—review & editing. CB: Conceptualization, Supervision, Writing—review & editing.

## Footnotes

^1^The purpose of this law is to ensure the right to equal opportunities and protect the social inclusion of autistic children, adolescents, and adults; eliminate any form of discrimination; promote a comprehensive approach to these people in the social, health, and education spheres and raises awareness in society about this issue. The foregoing, without prejudice to the other rights, benefits, or guarantees contemplated in other legislative bodies and in the international treaties ratified by Chile that are in force. This law contemplates a series of principles: Social Model of Disability, neurodiversity paradigm, dignity, progressive autonomy, gender perspective; intersectionality, participation, and social dialogue; early detection, and continuous monitoring (https://bcn.cl/3c7ek).
